# Supplementary figures and images for: Mis-splicing drives loss of function of p53E224D point mutation
Source: PLoS One. 2025 Mar 5;20(3):e0318856. doi: 10.1371/journal.pone.0318856 (PMC11882087; doi:10.1371/journal.pone.0318856)

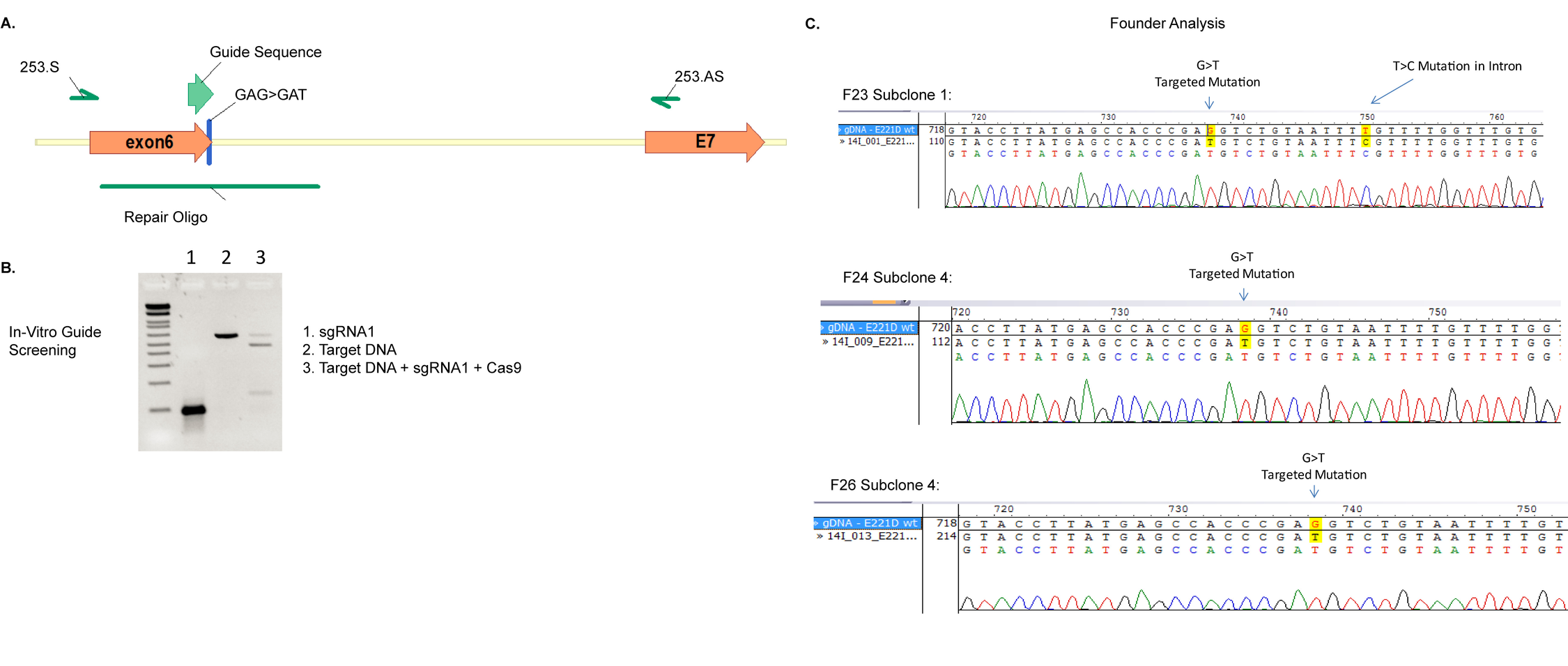

Supplement: S1 Fig — A) Targeting strategy for CRISPR mediated, homology-directed, repair of endogenous p53 gene for generation of p53 P53E221D/E221D embryos B) In-vitro guide screening of targeting construct C) Sanger analysis of three founder mice harboring the P53E221D/E221D mutation. (TIF) [file pone.0318856.s001.tif]

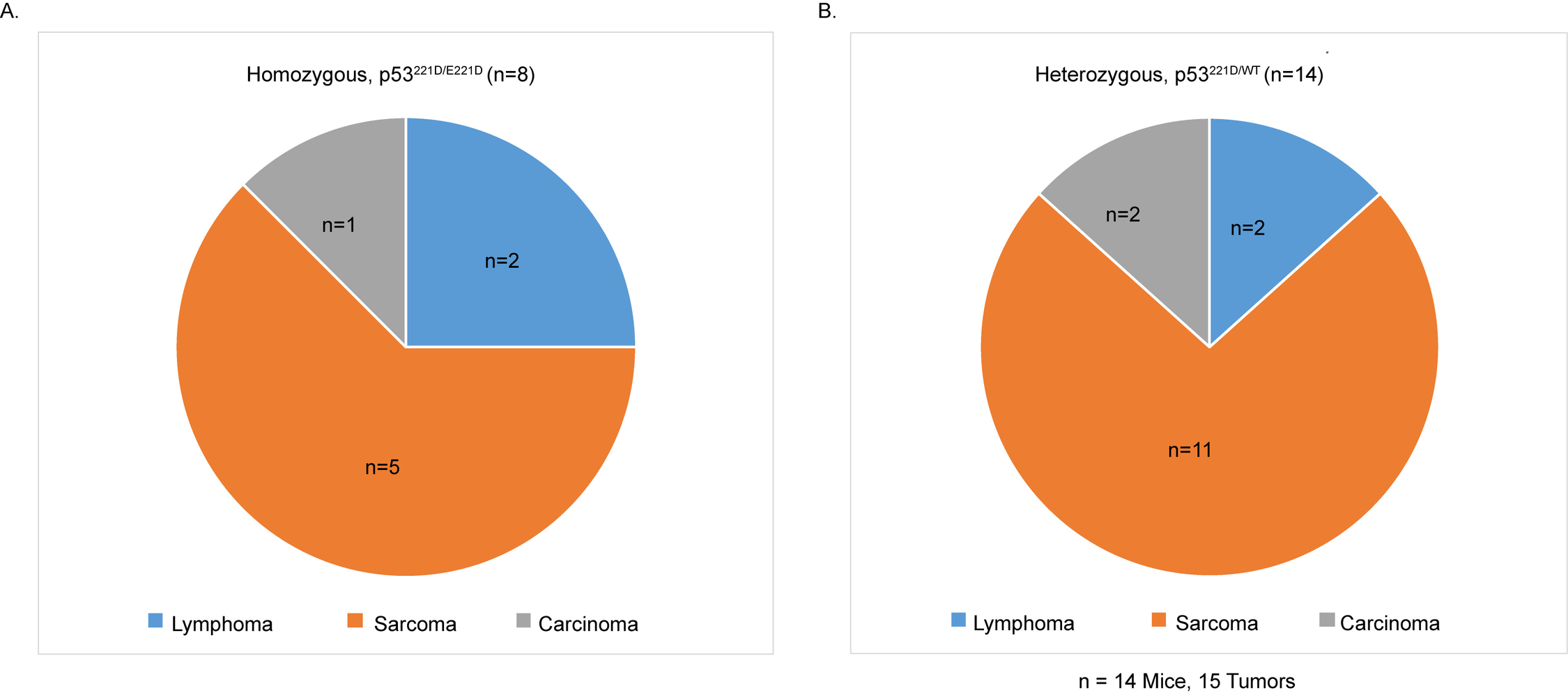

Supplement: S2 Fig — A) Pie chart of tumor spectrum observed in p53E221D/E221D littermate controls. B) Pie chart of tumor spectrum observed in p53E221D/WT littermate controls. Mouse 640847 presented multiple primary tumors, one lymphoma and one sarcoma and thus is counted in both categories. Note: Due to the constraints of COVID19 procedures, there was a reduced capacity for consistent mouse observation. This resulted in a reduced capacity for collection of recently deceased and a likely bias towards gross morphological tumors reaching humane endpoints and subsequent collection. Therefore, we provide these data as a qualitative indication of a conserved tumor spectrum rather than a quantitative assessment of subtype prevalence. Subtype prevalence was determined using H&E stained tumor sections and subsequent pathology assessment. (TIF) [file pone.0318856.s002.tif]

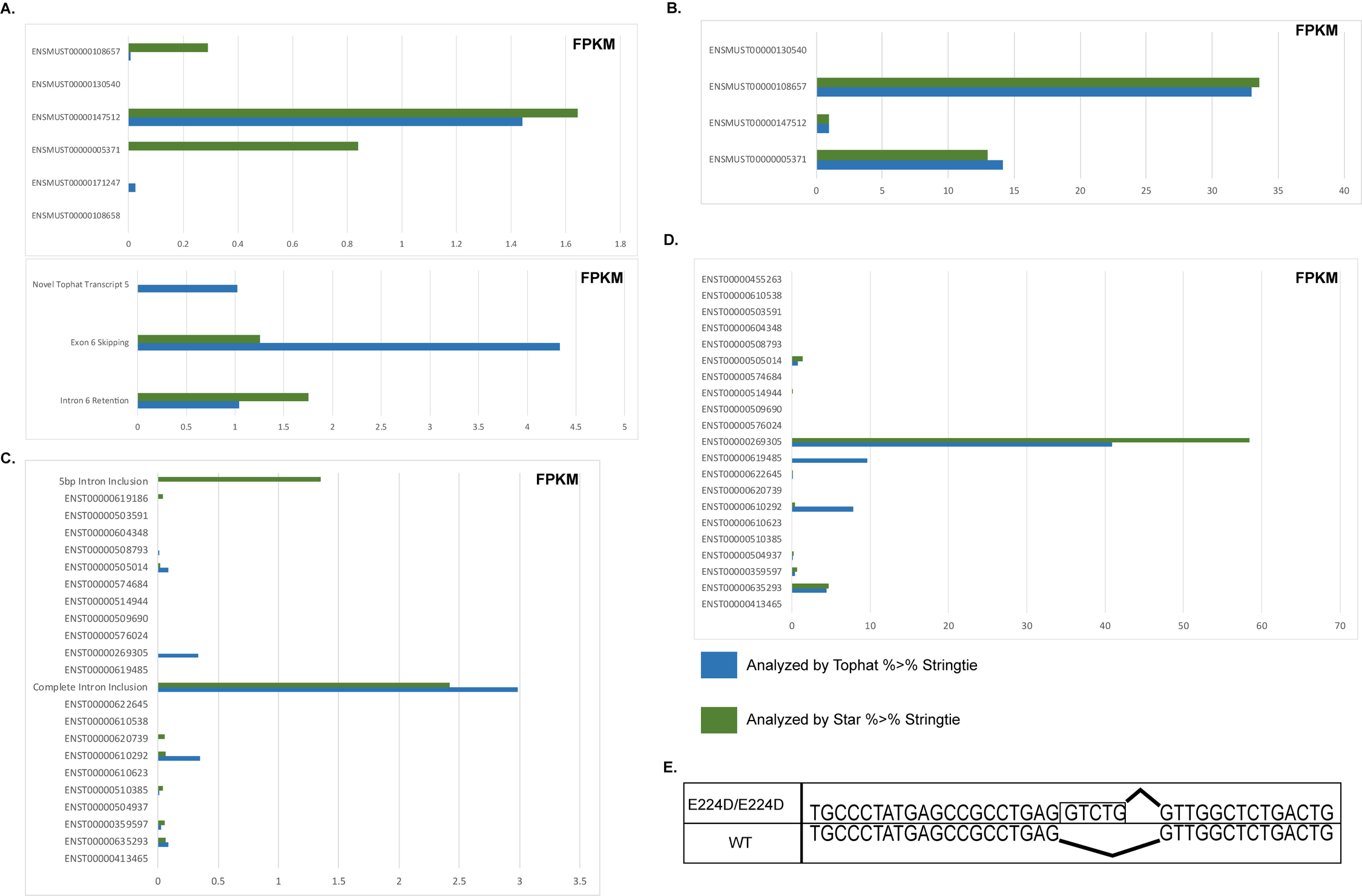

Supplement: S3 Fig — A) Bar chart comparison of known transcripts and novel splice events in Stringtie results of bam files aligned with Tophat and Star from p53E221D/E221D MEF samples. (Blue = Tophat aligned, Green = Star aligned) B) Bar chart comparison of known transcripts in Stringtie results from Tophat and Star aligned reads from p53WT MEF samples. (Blue = Tophat aligned, Green = Star aligned) C) Bar chart comparison of known transcripts and novel splice events in Stringtie results of bam files aligned with Tophat and Star from p53E224D/E224D NCI-H716 samples. Novel transcripts aggregated by the variation at the intron six 5’ splice donor site. (Blue = Tophat aligned, Green = Star aligned) D) Bar chart comparison of known transcripts in Stringtie results from Tophat and Star aligned reads from p53WT HCT116 samples. (Blue = Tophat aligned, Green = Star aligned) E) Cryptic splice site in novel transcript, labeled 5 bp intron inclusion in Supplementary Fig S3C, called in Stringtie results of Star alignments includes five base pairs of the intron in human cancer harboring the p53E224D/E224D. (TIF) [file pone.0318856.s003.tif]

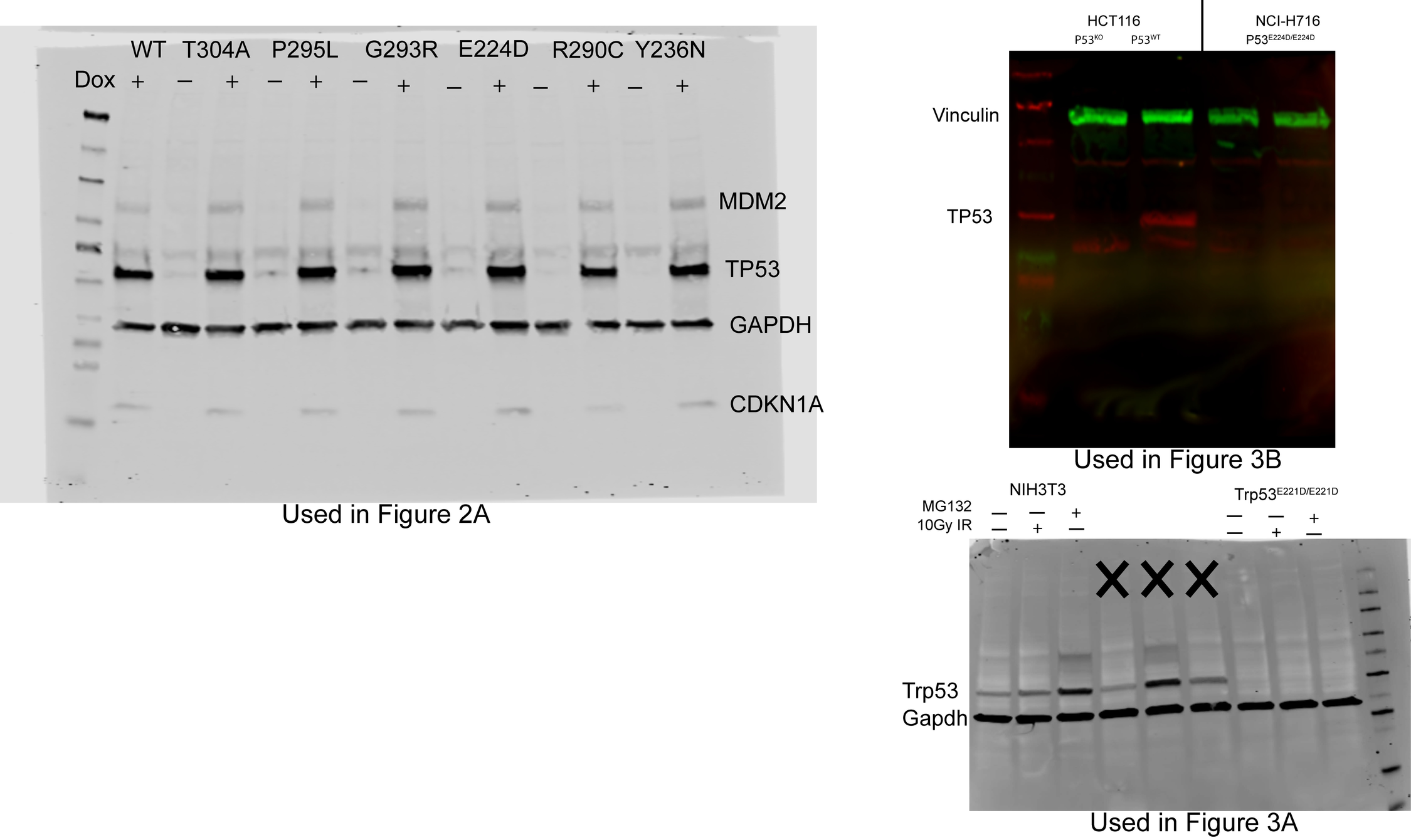

Supplement: S1 File — (TIF) [file pone.0318856.s005.tif]
