# Supplementary material for: Mis-splicing drives loss of function of p53E224D point mutation
Source: PLoS One. 2025 Mar 5;20(3):e0318856. doi: 10.1371/journal.pone.0318856 (PMC11882087; doi:10.1371/journal.pone.0318856)
Supplement: S1 Table — (DOCX) [file pone.0318856.s004.docx]

**S1 Table: Sequences used in Mouse Model Generation.**

| sgRNA | 5’ ggtaccttatgagccacccg 3’ |
| --- | --- |
| ssODN: 5’ ->3’ | Ccccagcatcttatccgggtggaaggaaatttgtatcccgagtatctggaagacaggcagacttttcgcc Acagcgtggtggtaccttatgagccacccga**t**gtctgtaattttgttttggtttgtgcgtcttagagacagt tgactccagcctagactgatgttgactttctagcaacccgtttgcctcaccctcctga |
| E221D-253S | 5’ ACCGCGGTGGCGGCCGC cttattcttgctcttaggcctggc 3’ |
| E221D-253AS | 5’ TAGAGGATCCACTAGT TGT AGT GGA TGG TGG TAT ACT CAG 3’ |
